# Cytogenetic findings in lung cancer that illuminate its biological history from adenomatous hyperplasia to bronchioalveolar carcinoma to adenocarcinoma: A case report

**DOI:** 10.3892/etm.2012.725

**Published:** 2012-09-27

**Authors:** DANIELA BETTIO, UMBERTO CARIBONI, ANNA VENCI, MARIALUISA VALENTE, PAOLA SPAGGIARI, MARCO ALLOISIO

**Affiliations:** 1 Cytogenetic and Medical Genetic Laboratory, Operative Unit of Clinical Investigations;; 2 Departments of Thoracic Surgery and; 3 Pathology, IRCCS Humanitas Clinical Institute, Rozzano, Milan, Italy

**Keywords:** lung cancer, ground-glass opacities, chromosome abnormalities, multi-step progression

## Abstract

The biological and chronological evolution of lung cancer remain to be fully elucidated. A multi-step carcinogenesis hypothesis suggests a progression from atypical adenomatous hyperplasia (AAH) through bronchioalveolar carcinoma (BAC) to invasive adenocarcinoma (AC), but to date this has not been formally demonstrated. We report a case of a patient diagnosed by computed tomography (CT) with lung cancer in the superior right lobe who also presented with a pure ground-glass opacity (GGO) in the inferior lobe, while the middle lobe appeared normal. Following pneumonectomy, cytogenetic analysis successfully performed on spontaneous metaphases obtained by the direct method from samples of the three lung lobes showed the presence of three clonal cell populations, each progressively having increased karyotype complexity. Fluorescence *in situ* hybridization (FISH), performed using *ALK* (2p23) break probe and *ALK/EML4* t(2;2);inv(2) fusion probe, showed a normal pattern for all specimens. Histological evaluation confirmed the presence of AC in the superior right lobe and classified the GGO lesion as BAC and the normal tissue of the middle lobe as AAH. To the best of our knowledge, this is the first case in which the cytogenetic study of spontaneous metaphases showed a clear clonal relationship among AC, BAC and AAH present simultaneously in different lobes of the same lung. This case appears to indicate that the entire lung was somehow predisposed to a neoplastic transformation starting with a diffuse AAH characterized by high proliferative activity. Moreover, the 5q13 region involved in the translocation shared by BAC and AC contains at least 4 genes encoding important regulators of the cell cycle that may be considered new molecular markers of lung cancer.

## Introduction

The pathogenesis of lung cancer and the criteria that regulate its progression are under investigation. Pulmonary lesions, such as small nodules with focal ground-glass opacity (GGO), have been increasingly detected due to the widespread use of computed tomography (CT) scanning. Histologically, these lesions can be classified as atypical adenomatous hyperplasia (AAH), bronchioalveolar carcinoma (BAC) or adenocarcinoma (AC). Several studies have suggested that AAH, frequently found in tissue surrounding lung AC, may be a forerunner in the development of AC; moreover, the more recent discovery of lung nodules manifesting as GGOs further supports a stepwise process in the development of pulmonary AC (1–3). However, the genetic relationship between AC and the associated foci of AAH is not yet well defined. In particular, it is not clear whether multiple foci of AAH and AC in the same patients are clonally related or are independent neoplastic foci (4). Several studies performed loss of heterozygosity (LOH), fluorescence *in situ* hybridization (FISH), microarrays and immunohistochemistry analyses and demonstrated an increasing genetic complexity associated with lung cancer progression (4–7) but, to the best of our knowledge, no cytogenetic study showing a clear clonal relationship among AC, BAC and AAH has been reported thus far.

We report the case of a patient histologically diagnosed with AC in the superior right lobe and BAC in the inferior lobe, previously identified as a pure GGO nodule by a CT scan. AAH was diagnosed in the middle lobe, considered to be normal at CT scan and during surgery. The cytogenetic studies performed on biopsies from the three lobes allowed the identification of different chromosome rearrangements with clonal evolution that supports the hypothesis of a complex multi-step carcinogenesis in which lung AC develops from AAH through BAC.

## Case report

**Figure 1:**
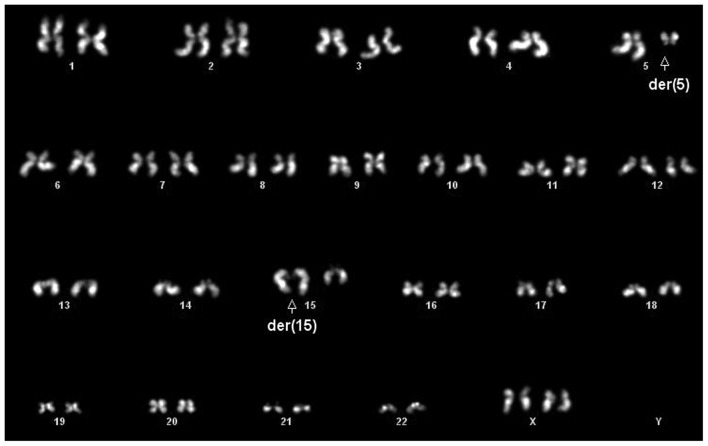
46,XX,t(5;15)(q13;q25-26) karyotype observed in the ground glass opacity lesion.

A 54-year-old female who never smoked was diagnosed with a lung tumor in the upper right lobe by a CT scan. A transthoracic fine-needle aspiration of the lesion was performed, followed by histological diagnosis of AC with a BAC component. The CT scan also showed a pure GGO lesion in the lower lobe, while the middle lobe appeared to be normal. The patient underwent pneumonectomy and samples from the 3 lung lobes were sent to the cytogenetic laboratory. Cytogenetic analyses were performed on spontaneous metaphases obtained by the direct method and short-term cultures as previously reported (8). The normal appearing middle lobe showed high spontaneous replication activity after 24 h of incubation, with 9 of 9 cells presenting a normal karyotype. The GGO lesion had 4 cells with a t(5;15)(q13;q25-26) as a single anomaly (Fig. 1), 2 cells with the t(5;15) translocation with complex rearrangements, including a derivative chromosome 1 with unknown additional material on the short arm, and 4 cells with a normal karyotype. In the AC, the same rearrangements present in the GGO were observed in 9 of 9 metaphases, 2 of which included the t(5;15). Most of the metaphases showing complex rearrangements were incomplete and in these cases we defined a composite karyotype following the ISCN recommendations based on the recurrent abnormalities observed (9): 44∼46,X,del(X) (p11.2),der(1)add(1)(p32),t(5;15)(q13;q25-26) [cp9].

The karyotypes obtained from all samples after short-term cultures (5–7 days) were normal, indicating that the tumor cytogenetic profile is rapidly obscured in short-term cultures due to a selective advantage of karyotypically normal cells, as previously reported (8).

Since a fusion gene between echinoderm microtubule-associated protein-like 4 (*EML4*) and anaplastic lymphoma kinase (*ALK*) has been identified in a subset of non-small cell lung cancer patients who never smoked (10), FISH was performed on our specimens using the *ALK* (2p23) break probe and *ALK/EML4* t(2;2); inv(2) Fusion Probe (Poseidon™, a gift from Kreatech Diagnostics, The Netherlands). A normal pattern was observed. This case report was part of a research project approved by the local ethics committee (authorization No. 850), and informed patient consent was obtained.

## Discussion

The molecular drivers that determine histology in lung cancer remain largely unknown and it is difficult to identify a valid parameter of tumor aggressiveness that may be used as a prognostic factor. The hypothesis of a multi-step carcinogenic process has recently been supported by the observations of Min *et al* (11) in a patient over a 10-year follow-up period, in whom CT and PET imaging findings showed the progression from a focal pure GGO nodule (presumed to be AAH or BAC) to an invasive AC.

The cytogenetic findings in our case support this hypothesis. The observation of active replication with spontaneous metaphases obtained after a few hours of incubation in the biopsy from the middle lobe suggests that in the normal appearing tissue the cell cycle control was lost. It is well known that cancer is a disease of hyper-proliferation predisposing to chromosome instability and this may have led to the first rearrangement we identified in the sample from the GGO, the translocation t(5;15)(q13;q25-26). Notably, the breakpoint at band q13 in the long arm of chromosome 5 is the same as we observed in a constitutional pericentric inversion previously reported in a patient with a pure GGO lesion (12). This band contains at least 4 genes (CCNB1, CDK7, CENPH, RAD17) encoding important regulators of the cell cycle that could be disrupted by the chromosome rearrangement (13). Moreover, a genome-wide association study reported that the chromosome 15q25.1 region, which includes three nicotinic cholinergic receptor genes (CHRNA5, CHRNB4, CHRN) and cell proliferation gene (PSMA4), is associated with lung cancer risk in Caucasian individuals irrespective of smoking status or propensity to smoke tobacco (14). In our case, the clone with the complex karyotype which was present in a few cells from the GGO and in all the cells from the AC probably had a strong proliferative advantage on the clones harboring the t(5;15). This is in agreement with the well-known observation that the complexity of chromosomal aberrations in cancer is correlated with the aggressiveness of the disease.

Common gene variants involved in lung cancer have been recently identified through large, collaborative, genome-wide association studies. Three loci markedly associated with lung cancer susceptibility have been reported: 5p15, 6p21 and 15q25, where genes that regulate acetylcholine niconitic receptors and telomerase production are located (15). In the present case, two of these three relevant regions were involved in chromosome rearrangements that may either cause gene inactivation or dysregulation, supporting their crucial role in the disease.

To the best of our knowledge, this is the first study to report a clonal relationship among AC, BAC and AAH, present simultaneously in different lobes of the same lung. This case suggests that the entire lung was somehow prone to the neoplastic transformation, possibly primed by cells with high proliferative activity such as those present in the middle lobe affected by AAH. Genetic studies of multiple lesions present in the same lung should be performed in order to verify this hypothesis.
